# Anti-Inflammatory and Cortical Responses after Transcranial Direct Current Stimulation in Disorders of Consciousness: An Exploratory Study

**DOI:** 10.3390/jcm13010108

**Published:** 2023-12-24

**Authors:** Sofia Straudi, Annibale Antonioni, Andrea Baroni, Valentina Bonsangue, Susanna Lavezzi, Giacomo Koch, Veronica Tisato, Nicole Ziliotto, Nino Basaglia, Paola Secchiero, Fabio Manfredini, Nicola Lamberti

**Affiliations:** 1 Department of Neuroscience and Rehabilitation, Ferrara University, 44121 Ferrara, Italy; strsfo@unife.it (S.S.); annibale.antonioni@edu.unife.it (A.A.); brnndr3@unife.it (A.B.); giacomo.koch@unife.it (G.K.); nino.basaglia@unife.it (N.B.);; 2 Department of Neuroscience, Ferrara University Hospital, 44124 Ferrara, Italy; v.bonsangue@ospfe.it (V.B.); s.lavezzi@ospfe.it (S.L.); 3 Doctoral Program in Translational Neurosciences and Neurotechnologies, Ferrara University, 44121 Ferrara, Italy; 4 Department of Translational Medicine, Ferrara University, 44121 Ferrara, Italy; 5 Department of Pharmacy, University of Pisa, 56126 Pisa, Italy; nicole.ziliotto@unipi.it

**Keywords:** disorders of consciousness, tDCS, fNIRS, biomarkers, inflammation

## Abstract

Disorders of consciousness (DoC) due to severe traumatic brain injury (TBI) are associated with severe disability and an alteration of cortical activation, angiogenesis, and inflammation, which are crucial elements for behavioural recovery. This exploratory study aimed to evaluate anti-inflammatory and cortical responses after transcranial direct current stimulation (tDCS) in traumatic prolonged disorders of consciousness. Ten minimally conscious state (MCS) patients underwent ten sessions of anodal tDCS (five sessions/week, two weeks, 40 min/session) on the primary motor cortex bilaterally. Clinical evaluations were performed using the Coma Recovery Scale–Revised (CRS-R) pre- and post-treatment. In contrast, after single and multiple tDCS sessions, the haemodynamic cortical response was obtained with functional near-infrared spectroscopy (fNIRS). Moreover, angiogenesis (angiopoietin-2, BMP9, endoglin, HbEFG, HGF, IL8, Leptin, PLGF, VEGF-A, and VEGF-C) and inflammation (GM-CSF, IFNg, IP10, MCP1, and TNFα) circulating biomarkers were collected. A significant haemodynamic response was observed after a single tDCS session, with an increased activation from 4.4 (3.1–6.1) to 7.6 (2.9–15.7) a.u. (*p* = 0.035). After ten tDCS sessions, a significant reduction of angiopoietin-2, VEGF-C, and IP-10 was detected. Moreover, a correlation between behavioural (CRS-R), TNFα (r = 0.89; *p* = 0.007), and IP10 (r = 0.81; *p* = 0.014) variation was found. In conclusion, a single tDCS session can increase the cortical activation in MCS patients. Moreover, multiple tDCS sessions showed an anti-inflammatory effect related to behavioural improvement.

## 1. Introduction

Severe brain injuries leading to disorders of consciousness (DOC) result in various impairments and have a substantial impact on public health costs. Vegetative state or persistent unresponsive wakefulness syndrome (VS/UWS) are characterised by a lack of overt behaviours indicating self- or environmental awareness [1], as opposed to the minimally conscious state (MCS), in which such signs are observable yet inconsistent [2]. Currently, there are no evidence-based recommendations available for treatments in DOC patients. Nevertheless, a promising intervention involves modulating the central nervous system’s activity by means of either pharmacological or brain-stimulation approaches [3], like transcranial direct current stimulation (tDCS) [4]. In the past decade, some clinical studies have explored the effects of tDCS in patients with prolonged disorders of consciousness with encouraging therapeutic outcomes [4]. For example, a recent study proved that treatment with anodal tDCS applied to the left dorsolateral prefrontal cortex (DLPFC) for 20 min in patients with MCS due to severe brain damage could lead to a brief improvement in consciousness [5]; moreover, Angelakis et al. also highlighted an improvement after stimulation over the left DLPFC in three of the ten recruited patients [6]. Moreover, some indications of DOC patients who might benefit more from tDCS have been provided, revealing how MCS with traumatic aetiologies respond more consistently to this therapeutic approach [7]. However, despite promising behavioural effects, no firm conclusions can be made due to the heterogeneity of methodologies, brain lesions, and low sample size.

In this study, we stimulate the primary motor cortex (M1) bilaterally with anodal tDCS, considering M1 as a potential entry door to subcortical structures, such as the central thalamus, which has a central role in arousal regulation. Previously, it has been demonstrated how central thalamic stimulation with deep brain stimulation (DBS) increased behavioural awareness in severe brain injury patients [8].

The main mechanisms underlying the effects of M1 tDCS are the ability to modulate the brain cortex excitability based on the applied current’s polarity both during and after stimulation, and various functional connectivity patterns between the cortical and subcortical networks [9,10]. Specifically, a recent study by Aloi et al. showed that tDCS applied on M1 can influence thalamocortical coupling indirectly by targeting the surface (and, thus, easily accessible) regions in the motor network [11]. The role of thalamocortical oscillators in the genesis of the state of consciousness is well-established [12]. Furthermore, considering the crucial role of M1 in the motor network, NIBS techniques could improve patients’ motor performance in DOC patients and, thus, their ability to interact with the environment [13]. It is known that some MCS patients highlight neurophysiological and neuroimaging responses consistent with residual consciousness [14], but cannot show them due to limited motor capacity. For these reasons, even if it is not directly part of the network of arousal and awareness, M1 can be considered a promising target for NIBS techniques in DOC patients. However, a single session of tDCS on M1 failed to improve the behavioural responsiveness in patients with DOC [13]. This could be due to the low behavioural effect of a single session or the need to stimulate M1-connected networks more extensively to highlight an overt improvement. A previous study showed increased Coma Recovery Scale–Revised (CRS-R) scores after ten sessions of bilateral M1 anodal tDCS in MCS [15].

Beyond the effects on the neural excitability, the modulation of the regional cerebral flow and cerebral vasomotor reactivity have been demonstrated after tDCS [16,17]. Cerebral blood flow (CBF) supplying glucose through neurovascular coupling is closely linked to neural activity. Notably, functional near-infrared spectroscopy (fNIRS) provides an excellent option to examine the cerebral oxygenation and blood volume, i.e., the haemodynamic response [18]. This technique offers spatial and temporal resolution for brain activity near the cortical surface. Although tDCS–NIRS studies are in their infancy, it has been found that, in healthy subjects, prefrontal and sensorimotor anodal tDCS determines a focal increase in the HBO_2_ concentration in the area under the electrode [19,20]. Some studies are available using fNIRS, focusing on patients with DOC, supporting an increasing interest in this technique. So far, this field’s research has focused on detecting cerebral activity at rest or during somatosensory stimulation and motor-imagery tasks [21,22].

Moreover, emerging evidence suggests that several other cellular and molecular mechanisms may contribute to the effects of tDCS, related to the modulation of angiogenesis, neurogenesis, and inflammatory response [23,24]. Considering the bidirectional link between inflammation and angiogenesis, and their crucial role in recovery after brain damage of various kinds, these aspects also seem essential to DOC patients, but, surprisingly, available data are scarce and often controversial [25,26,27].

Thus, we chose to evaluate the effects of bilateral M1 tDCS in terms of haemodynamic changes after a single session, and inflammation and angiogenesis after ten sessions. In this framework, we can expect that adding peripheral biomarkers and neuroimaging assessment would be helpful to personalise the neuromodulation intervention. The aims of this study were two-fold: (i) exploring haemodynamic neural changes, measured by fNIRS, after one and multiple sessions of bilateral M1 anodal tDCS in patients with chronic MCS; (ii) evaluating the after-effects of multiple tDCS sessions in circulating biomarkers of angiogenesis and inflammation in relation with behavioural changes. Among DOC patients, we focused on MCS patients because they are generally characterised by a better prognosis compared with VS/UWS patients, who reflect more severe brain damage that limits spontaneous or pharmacologically induced recovery [28].

## 2. Materials and Methods

### 2.1. Participants

We conducted a longitudinal pilot study at the Severe Brain Injury Unit (Neuroscience Department, Ferrara University Hospital, Italy), recruiting MCS patients admitted for a multidisciplinary rehabilitation program due to traumatic brain injury (TBI), between November 2014 and October 2016. All the procedures were conducted according to the ethical standard of the Declaration of Helsinki [29]. The University Hospital of Ferrara, Italy, ethics committee approved the study, which was thus registered on the Clinicaltrial.gov database (ID protocol: NCT02288533). The eligibility of each MCS patient was verified by means of a screening procedure and, for each enrolled one, written informed consent was obtained from the legal representative after the latter and physicians had been informed about the procedures and purposes of the study. Specifically, inclusion criteria comprised: (i) men and women between 18 and 70 years; (ii) diagnosis of the MCS DOC stage, as assessed by CRS-R [30]; (iii) traumatic aetiology; (iv) at least one year after the injury. Exclusion criteria were: tDCS contraindications, such as ferromagnetic implants, which can be stimulated, misplaced, or over-heated because of the applied current, skull defects or skull plates, and relevant medical diseases (e.g., severe renal, cardiac, or hepatic insufficiency). For the baseline comparison between circulating biomarkers, eight age- and sex-matched healthy controls were also enrolled.

### 2.2. Intervention

Enrolled patients underwent ten sessions (five sessions/week for two weeks) of anodal tDCS. Specifically, we applied tDCS by means of a battery-driven constant-current stimulator (Brainstim, EMS, Bologna, Italy) consisting of two anodal electrodes (anode) on bilateral M1, while the cathode (reference electrode) was placed at the nasion. The electrode sponge, with a surface area of 16 cm^2^ (4 cm × 4 cm), was soaked in a saline solution. Each session involved the application of tDCS for 40 min at a current intensity of 2 mA. At the conclusion of each session, participants completed an adverse-event questionnaire related to tDCS, and investigators documented any observed behavioural changes. Skin redness or lesions and clinical signs of discomfort were evaluated.

### 2.3. Outcomes

#### 2.3.1. Clinical and Behavioural Assessments

**Figure 1:**
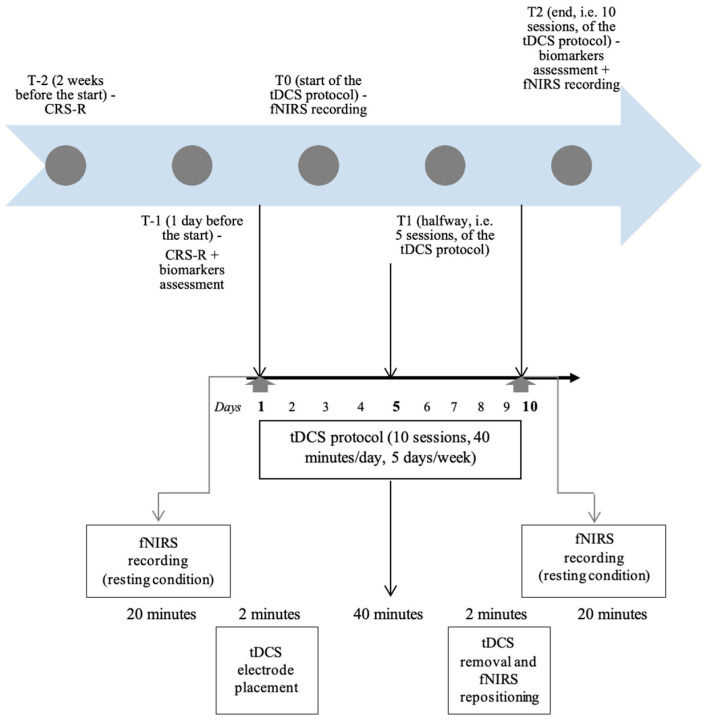
Timeline summary of the study protocol.

We carried out clinical evaluations using the Italian version of the CRS-R, a standardised neurobehavioural scale aimed at assessing the residual functions of DOC patients [30,31]. It comprises twenty-nine hierarchically organised items divided into six subscales evaluating visual, auditory, verbal, communication, motor, and arousal functions. Two experienced clinicians (a physician and a physiotherapist) with specific training to administer the scale assessed every patient. The CRS-R was administered two weeks before (T-2) and one day before (T-1) the start of the experimental protocol, halfway through (after five sessions) (T1), and at the end of the ten sessions (T2). The study timeline has been summarised in Figure 1.

#### 2.3.2. fNIRS Data Acquisition and Analysis

The haemodynamic signals were collected from the optical changes registered by means of a continuous wave, an fNIRS system (NIRScout, NIRx Medical Technologies, Glen Head, NY, USA) consisting of 16 LED lighting generators emitting two wavelengths of near-infrared light (760 and 850 nm), and 16 optical detectors, with a sampling rate of 3.47 Hz. Sources and detectors were placed on the measuring cap with reference to the 10–20 international system [32]. On the cap, the optodes’ spatial distribution was chosen to result on channels (i.e., source–detector pairs) with standard interoptode distances of approximately 30 mm. Optode placement was set to the sensorimotor areas of both hemispheres for 24 channels each. The fNIRS headpiece was secured on the forehead and held securely under the chin with an elastic strap. After the NIRS positioning, a 20 min recording time in resting conditions was carried out. Then, tDCS stimulation was performed according to the design described in the previous section for 40 min. Within 3 min from the end of the tDCS stimulation, the fNIRS headpiece was repositioned in the same spot, and another 20 min acquisition period was carried out. The experimental design lasted for a total of 85 min. The fNIRS recording was performed on the first day (T0) of the tDCS and the last (T2) day of brain stimulation.

The raw intensity data were analysed offline using the NIRSLab software version 2019.04. The optical signals of each channel were converted to oxygenated haemoglobin (oxy-Hb) and deoxygenated haemoglobin (deoxy-Hb) concentration changes by using the modified Beer–Lambert equation [33]. The software checked the data quality for each of the 48 channels, the resulting ‘bad’ channels were automatically removed, and the spike artefacts were corrected. If the total number of channels with a too-high signal-to-noise ratio was greater than 8 per hemisphere, that patient was excluded from further analyses. The final oxy-Hb track for each channel was plotted in an electronic sheet, and the area under the curve (AUC) was calculated by summing up the single oxygenation values for the total 20 min [34]. The resulting parameter, O_2_Hb_AUC_, was calculated for each recorded session.

#### 2.3.3. Circulating Biomarkers

Considering the modulatory effect of tDCS on angiogenesis and inflammation [35,36,37], and the prognostic role of circulating biomarkers in traumatic brain injury patients [38], we used a comprehensive biomarkers panel of 15 molecules related to angiogenesis; specifically, angiopoietin-2, bone morphogenetic 9 (BMP9), endoglin, heparin-binding EGF-like growth factor (HbEFG), hepatocyte growth factor (HGF), interleukin 8 (IL8), leptin, placental growth factor (PLGF), vascular endothelial growth factor-A and C (VEGF-A and VEGF-C), and inflammation, i.e., granulocyte–macrophage colony-stimulating factor (GM-CSF), interferon-gamma (IFNg), interferon-γ–inducible protein 10 (IP10), monocyte chemoattractant protein-1 (MCP1), and tumour necrosis factor α (TNFα), to evaluate their role and to detect any changes induced by the tDCS treatment. Thus, the circulating biomarkers were assessed before the start (T-1) and at the end (T2) of the tDCS protocol. Notably, these biomarkers were also measured at T-1 on a sample of 8 healthy controls to detect any differences present at baseline in the MCS patients.

Soluble biomarkers have been quantified using Milliplex MAP kits (Luminex xMAP technology, Merck-Millipore, Darmstadt, Germany). Samples were processed following the manufacturer’s recommended protocols and read on a MAGPIX instrument equipped with the MILLIPLEX-Analyst 5.1 Software (Merk Millipore, Darmstadt, Germany) using a five-parameter nonlinear regression formula to compute sample concentrations from the standard curves.

### 2.4. Statistical Analysis

For CRS-R, we considered the average of the data obtained at T-2 and T-1 as the baseline, while for the fNIRS and circulating biomarkers, we considered T0 and T-1 as the baseline, respectively. Data distribution was verified through a Shapiro–Wilk test. Due to the non-normal distribution, nonparametric tests were employed. The comparison between the diseased patients and healthy controls was performed via the Mann–Whitney test. Moreover, a repeated-measures analysis (Friedman test) with a post hoc test was carried out to explore any possible modification of the CRS-R scores, fNIRS data, and circulating biomarkers across time. All pair-wise comparisons were calculated with the Newman–Keuls post hoc test. We estimated the effect sizes through the partial eta square measure (ηp^2^). Correlations between parameters were verified through a Spearmans’ rho. We performed statistical analysis using STATA 13.1 software and showed a significance when *p* < 0.05.

## 3. Results

We enrolled ten patients (35.5 ± 12.6 years, seven males and three females, 5.5 ± 5.4 years post-trauma). Demographic and clinical characteristics of the sample are reported in Table 1.

### 3.1. Brain-Based Haemodynamic Changes after Bilateral M1 Anodal tDCS

The fNIRS data of the ten patients included were analysed. Unfortunately, three patients were excluded due to the high number of channels with a high signal-to-noise ratio (ID 5, 6, 7). The remaining seven patients completed all the tDCS sessions scheduled.

### 3.2. Single-tDCS-Session Effect

**Figure 2:**
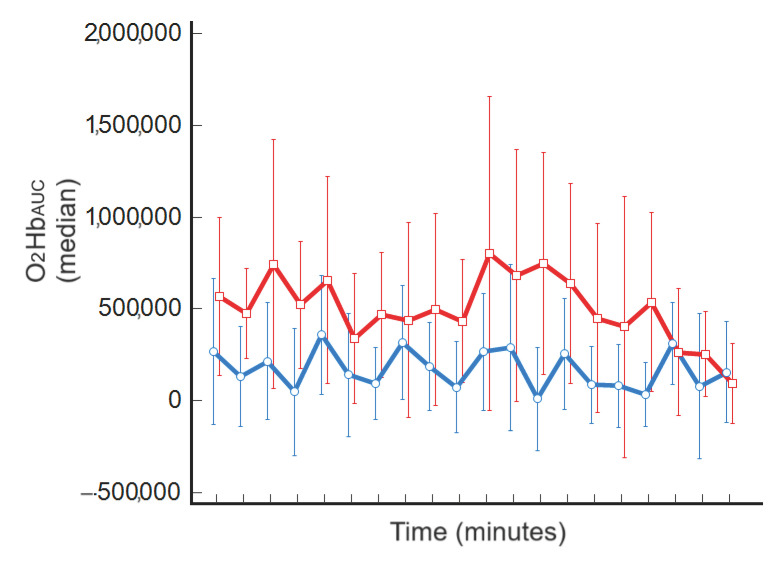
Median values of O_2_Hb_AUC_ before (blue line) and after tDCS (red line) (minutes on the *x*-axis).

A significant haemodynamic single-tDCS-session effect was observed in the seven subjects, with O_2_Hb_AUC_ median values exhibiting a significant increase after stimulation, from 4.4 (3.1–6.1) to 7.6 (2.9–15.7) a.u. (*p* = 0.035). A graphical representation of the cortical activation obtained for each minute of recording before and after tDCS is reported in Figure 2.

### 3.3. Multiple-tDCS-Sessions Effect

No significant haemodynamic effects were observed during the baseline recording from the first to the tenth tDCS sessions, with almost stable O_2_Hb_AUC_ values from 6.8 (2.1−8.9) to 7.0 (1.7−12.3) a.u. (*p* = 1.00). Interestingly, three subjects showed a progressive increase in O_2_Hb_AUC_ values (ID 2, 4, 9) over time, but no significant correlations with CRS-scale variation were observed.

### 3.4. Circulating Biomarkers

A panel of fifteen circulating biomarkers of angiogenesis and inflammation were analysed at baseline and the end of the tDCS sessions. Table 2 summarises the circulating biomarkers measured at T-1, comparing healthy controls (N = 8) and MCS patients. Significant differences were observed for IL-8, endoglin, leptin, GM-CSF, IFNg, IP10, and MCP1. After the study protocol, significant differences were observed in the MCS patients for angiopoietin-2, VEGC-F, and IP-10. In general, the signals of promotion of angiogenesis and the inhibition of inflammatory factors were observed. Data are reported in Table 3.

### 3.5. Relation between Biomarkers and Clinical Parameters

**Figure 3:**
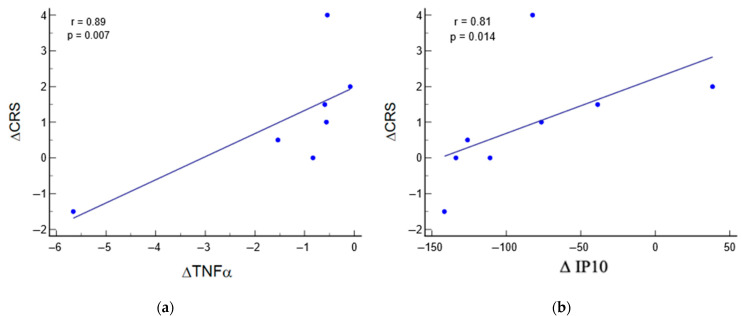
Correlation between variation in CRS and variation in TNFα (**a**) and IP10 (**b**). CRS, Coma Recovery Scale; r, correlation coefficient; p, significance.

Significant correlations were observed between the variations in CRS at the end of the multiple tDCS sessions and the two biomarkers belonging to the inflammatory panel: TNFα (r = 0.89; *p* = 0.007) and IP10 (r = 0.81; *p* = 0.014) (Figure 3).

## 4. Discussion

In the present study, traumatic prolonged disorders of consciousness patients received a multimodal assessment to determine the underlying mechanisms associated with behavioural effects after the tDCS. Specifically, the potential effects of the tDCS on cortical activity and inflammation were tested. A short-lasting (single session) effect on motor cortical activation has been found, whereas modifications after multiple sessions were anecdotally reported in our sample. Moreover, multiple tDCS sessions were related to an overall reduction in inflammation processes and behavioural changes over time.

In the last decade, fNIRS has gained popularity for assessing brain disorders, including DOC patients. It is a noninvasive, portable, and tolerable technique promising for bedside functional neuroimaging, even with technical challenges for data collection. So far, few studies investigated residual awareness in DOC patients during motor and sensory tasks [39], motor imagery tasks [40], or a resting state [21]. However, no studies are available to detect the cortical activity variation after tDCS, which has been proven effective in specific DOC phenotypes to increase awareness [7].

Indeed, in recent years, NIBS techniques have been promising in the treatment of various pathological conditions that require the modulation of brain networks, e.g., depression and substance addiction [41,42]. Considering that they are safe and well-tolerated, interest in their application is growing, particularly in pathological contexts, where usual treatments are ineffective or even unavailable [43]. NIBS techniques have consistently been used in DOC patients, with encouraging results. However, studies are still relatively limited and further evidence is needed to confirm their effectiveness, considering these conditions’ extreme heterogeneity in aetiology and clinical severity [28,44]. For example, Thibaut et al. applied a tDCS protocol on the left DLPF cortex in MCS and VS/UWS patients, showing a significant improvement, as assessed by CRS-R scores, only in the former, but not in the latter [5]. Consistently, a recent systematic review and meta-analysis on the efficacy of NIBS techniques for DOC highlighted that they are able to improve consciousness in MCS patients, but not in VS/UWS ones [45]. Indeed, the former condition is generally associated with a better prognosis than the latter, which is often due to more severe encephalic damage and is, therefore, less amenable to spontaneous or pharmacologically induced improvement [28]. Consequently, based on the evidence already reported in the literature, we focused on MCS patients, assuming they might have a better chance of recovery in association with the tDCS treatment.

After a single tDCS session applied bilaterally over the primary motor cortex, we observed a short-lasting increase in O_2_Hb_AUC_ in the motor area. In contrast, no effects were reported at the end of the treatment (10 sessions) or concerning behavioural improvements. This mismatch can be explained by the fact that the prefrontal cortex, instead of the motor cortex, has been linked to the awareness brain network [2], and that the increased motor cortical activation immediately after the tDCS reflected short-lasting effects under the area of stimulation instead of a structural change in brain connectivity. The results are consistent with previous studies conducted on healthy subjects that found transient changes in the haemodynamic profile after a single tDCS session [20,46]. Moreover, these focal changes in brain haemodynamics can help define the spatial resolution of the neuromodulation dosage [47] and evaluate the impact of the tDCS on cortical function.

In addition to the neuroimaging assessment, we investigated the peripheral variation in serum biomarkers after tDCS, leading to the hypothesis that a proinflammatory response was activated even months or years after the severe traumatic brain injury [48]. Indeed, the prognosis of DOC is still very complex, and there is growing interest in the role of biomarkers available on blood and cerebrospinal fluid (CSF) [25]. To better characterise the pathophysiology of DOC and to identify potential biomarkers of severity and prognosis, numerous preclinical animal models (e.g., head-open and head-closed injury models) have been developed and, among the many aspects assessed (e.g., behavioural, neuroimaging), a great deal of interest is directed towards biomarkers [49]. In particular, nonspecific biomarkers, including those for damage (autophagy or apoptosis), inflammation (proinflammatory and anti-inflammatory cytokines), metabolic changes, and degeneration (nerve growth factor, vascular endothelial growth factor, tau protein) seem very promising [50]. While a fair amount of data are available on these animal models, the evidence on humans is still inconclusive due to the extreme heterogeneity that characterises DOC patients. A recent study by Musso et al. showed a good potential for microRNAs, whereas the role of neuroinflammatory and angiogenetic biomarkers is still quite controversial [51]. Of note, after a severe TBI, a neural inflammation is triggered by the activation of several biological processes that can be sustained by the prolonged release of cytokines [52]. This biological response is usually related to the worst functional outcomes. Indeed, prolonged disorders of consciousness had significantly higher levels of IL-8, leptin, and IP-10, and a reduction in INFg compared with healthy subjects. Higher leptin levels have been associated with unfavourable outcomes after paediatric traumatic brain injury [53].

Similarly, higher levels of IL-8, a proinflammatory cytokine, have been associated with higher mortality and poor outcomes in the acute phase [52]. Confirming this evidence, a recent study of patients affected by TBI in the acute phase (within 12 h after TBI) showed that elevated levels of caspase-1 (an inflammasome protein) and IL-10 (a proinflammatory cytokine) could predict an unfavourable outcome [54]. It thus seems that the acute activation of specific inflammation pathways correlates with an unfavourable outcome, probably reflecting more severe damage and/or impairment in repair processes. Interestingly, Licastro et al. focused on the chronic TBI phase and showed that persistent high plasma levels of cytokines could interfere with cognitive functioning, and that higher postacute levels of cytokines (i.e., IFN-γ, TNF-α, IL1b IL6) were associated with poorer cognitive recoveries 12 months later [55]. Furthermore, a recent study highlighted that IL-13 and TNF-α correlated with behavioural scores in prolonged DOC, and that they were associated with recovery 12 months later, suggesting a potential prognostic role of peripheral biomarkers [26]. However, data on this regard are still conflicting, and more evidence is required about their prognostic significance.

Moreover, considering the evidence linking inflammation and angiogenesis in a complex bidirectional relationship, and the role of the latter in recovery after brain damage of various kinds, it seemed reasonable to investigate the prognostic potential played by angiogenesis in DOC to explore this topic, which is still poorly explored in the literature [56,57]. Indeed, it has been shown that tDCS could improve poststroke recovery by also modulating neuroinflammation and angiogenesis in the central nervous system, and interest is also growing in other pathological conditions [58]. Consistently applying multiple tDCS sessions can potentially modulate these biological cascades, determining a significant reduction in angiopoietin-2 and IP-10, for example. The role of tDCS in neuroinflammation has been previously addressed in animal models [59,60] and other neurological conditions, such as bipolar disorders [35], leading to the hypothesis that tDCS can influence cytokine release in the brain and reduce neural inflammation. Here, importantly, we showed that some biomarkers, i.e., TNFα and IP-10, directly correlate with behavioural changes after the tDCS, as measured by the CRS-R scale. These findings confirm the evidence on the potential long-term prognostic role of circulating biomarkers of inflammation and angiogenesis, opening the window toward new biological targets to evaluate the responsiveness to tDCS in prolonged disorders of consciousness.

### Limitations

This exploratory study with a small sample size and a lack of a control group prevents us from drawing definitive conclusions on the cortical and inflammatory response after the tDCS in MCS. These preliminary findings should be further investigated in a larger sample size with a control group to confirm the role of the tDCS in modulating cortical activity and reducing inflammation in prolonged disorders of consciousness. Moreover, interindividual variability due to different anatomic features and brain lesions could have masked the tDCS effects [61], leading to the conclusion that a tailored neuromodulation approach would be necessary for the forthcoming studies. Furthermore, the decision to exclude, due to reduced quality of the fNIRS data, three patients from the analyses further reduced our limited sample, preventing the generalisation of the results. Finally, the application of NIBS in DOC has several ethical concerns, considering that the informed consent is given by a legal representative and not by the patient.

## 5. Conclusions

In prolonged disorders of consciousness, the application of the tDCS had a short-lasting effect on cortical activity under the area of stimulation, without any significant long-lasting effects. Moreover, in these patients, an overall increase in inflammatory biomarkers has been found, along with a reduction in inflammation after multiple tDCS sessions. Clinical effects can be linked to reduced inflammatory biomarkers, such as TNFα and IP-10. This exploratory study proposed a new multimodal approach for managing prolonged disorders of consciousness to personalise and improve their treatment.

## Figures and Tables

**Table 1:** Characteristics of the sample.

Patient ID	Sex	Age	Time Since TBI	Medications	Implantable Devices	CRS-R Baseline
1	M	35	11 y	-	ITB	15
2	M	36	8 y, 9 m	-	VPS	11
3	M	47	4 y, 7m	Levetiracetam	ITB	13
4	M	34	19 y	-	-	12
5	F	24	2 y	Levetiracetam, Amantadin	-	11
6	F	27	7 y, 6 m	Levetiracetam	ITB	9
7	M	42	2 y, 7 m	-	VPS	9
8	M	26	1 y, 3 m	Lamictal, Fenobarbital	-	9
9	M	63	1 y	Carbamazepine	VPS	8
10	M	21	1 y, 1 m	-	VPS	9

M, male; F, female; TBI, traumatic brain injury; y, years; m, months; ITB, intrathecal baclofen; VPS, ventriculoperitoneal shunt; CRS-R, Coma Recovery Scale–Revised.

**Table 2:** Comparison between circulating biomarkers in the MCS patients and healthy subjects at T-1.

	Healthy Subjects (N = 8)	MCS Patients (N = 10)	*p* Value
Angiopoietin-2	1616 (1121–2111)	1870 (1140–2601)	0.514
BMP9	228 (155–301)	174 (86–262)	0.288
Endoglin	931 (720–1141)	553 (332–773)	0.011
HbEFG	32 (20–45)	26 (15–36)	0.387
HGF	181 (145–217)	247 (167–327)	0.180
IL8	3.3 (2.6–4.1)	10.5 (0.1–22.2)	<0.001
Leptin	5850 (2392–9308)	16,590 (7697–25,482)	0.027
PLGF	N.D.	11.6 (4.1–19.1)	N.A.
VEGF-A	N.D.	121 (15–258)	N.A.
VEGF-C	300 (212–387)	330 (259–401)	0.545
GM-CSF	9.89 (5.59–14.19)	3.49 (1.10–5.89)	0.011
IFNg	10.15 (0.37–24.07)	5.58 (2.70–8.47)	<0.001
IP10	222 (153–292)	507 (61–953)	<0.001
MCP1	281 (234–328)	373 (223–523)	0.008
TNFα	2.97 (1.52–4.43)	4.03 (1.61–6.45)	0.39

BMP9: bone morphogenetic protein 9; HbEFG: heparin-binding EGF-like growth factor; HGF: hepatocyte growth factor; IL8: interleukin 8; PLGF: placental growth factor; VEGF-A and -C: vascular endothelial growth factor-A and C. GM-CSF: granulocyte–macrophage colony-stimulating factor; IFNg: interferon-gamma; IP10: interferon-γ–inducible protein 10; MCP1: monocyte chemoattractant protein-1; TNFα: tumour necrosis factor α. Protein levels are reported in pg/mL (median (IQR)). N.D.: not detectable, protein levels were below the limit of detection. N.A.: not available. *p*-values refer to the median (IQR).

**Table 3:** Circulating biomarkers before and after tDCS.

	Day 1	Day 10	*p* Value
Angiopoietin-2	1870 (1140–2601)	1535 (955–2113)	0.040
BMP9	174 (86–262)	185 (104–265)	0.39
Endoglin	553 (332–773)	585 (369–800)	0.22
HbEFG	26 (15–36)	36 (19–52)	0.14
HGF	247 (167–327)	234 (155–312)	0.33
IL8	10.5 (0.1–22.2)	11.0 (2.3–24.0)	0.66
Leptin	16,590 (7697–25,482)	14,682 (7155–22,210)	0.060
PLGF	11.6 (4.1–19.1)	10.6 (1.6–19.5)	0.56
VEGF-A	121 (15–258)	130 (22–247)	0.73
VEGF-C	330 (259–401)	388 (307–469)	0.041
GM-CSF	3.49 (1.10–5.89)	4.40 (2.17–6.61)	0.30
IFNg	5.58 (2.70–8.47)	5.16 (1.75–8.57)	0.75
IP10	507 (61–953)	423 (1–846)	0.006
MCP1	373 (223–523)	355 (236–474)	0.56
TNFα	4.03 (1.61–6.45)	2.63 (1.63–3.62)	0.10

BMP9: bone morphogenetic protein 9; HbEFG: heparin-binding EGF-like growth factor; HGF: hepatocyte growth factor; IL8: interleukin 8; PLGF: placental growth factor; VEGF-A and -C: vascular endothelial growth factor-A and C. GM-CSF: granulocyte–macrophage colony-stimulating Factor; IFNg: interferon-gamma; IP10: interferon-γ–inducible protein 10; MCP1: monocyte chemoattractant protein-1; TNFα: tumour necrosis factor α. Protein levels are reported in pg/mL (median (IQR). *p*-values refer to the Newman–Keuls post hoc test.

## Data Availability

The data supporting this study’s findings are available from the corresponding author upon reasonable request.
